# Intelligence, creativity, and cognitive control: The common and differential involvement of executive functions in intelligence and creativity

**DOI:** 10.1016/j.intell.2014.05.007

**Published:** 2014-09

**Authors:** Mathias Benedek, Emanuel Jauk, Markus Sommer, Martin Arendasy, Aljoscha C. Neubauer

**Affiliations:** Department of Psychology, University of Graz, Austria

**Keywords:** Intelligence, Creativity, Divergent thinking, Executive control, Working memory

## Abstract

Intelligence and creativity are known to be correlated constructs suggesting that they share a common cognitive basis. The present study assessed three specific executive abilities – updating, shifting, and inhibition – and examined their common and differential relations to fluid intelligence and creativity (i.e., divergent thinking ability) within a latent variable model approach. Additionally, it was tested whether the correlation of fluid intelligence and creativity can be explained by a common executive involvement. As expected, fluid intelligence was strongly predicted by updating, but not by shifting or inhibition. Creativity was predicted by updating and inhibition, but not by shifting. Moreover, updating (and the personality factor openness) was found to explain a relevant part of the shared variance between intelligence and creativity. The findings provide direct support for the executive involvement in creative thought and shed further light on the functional relationship between intelligence and creativity.

## Highlights

• Latent variable analysis relating executive functions, intelligence and creativity
• Intelligence is predicted by updating, but not inhibition or shifting ability.
• Creativity is predicted by updating and inhibition, but not shifting ability.
• Updating explains a relevant part of the intelligence–creativity relationship.

## 1 Introduction

Intelligence and creativity are correlated constructs, but what is the reason for this relationship? One possible explanation would be the common involvement of similar executive processes. Executive functions contribute to the performance in complex cognitive tasks, and are thought to represent the elementary cognitive basis of individual differences in general intelligence. There is also increasing evidence that EFs are highly relevant for creativity. However, it remains unclear what specific EFs are actually involved. The present study hence aims to investigate the common or differential role of three EFs (i.e., updating, shifting, and inhibition) for intelligence and creativity, and to clarify whether EFs can partly explain the correlation between intelligence and creativity.

### 1.1 The relationship of intelligence and creativity

Creativity is commonly defined by novelty and usefulness (Barron, 1955, Stein, 1953; cf. Runco & Jaeger, 2012). This definition applies to the evaluation of single pieces of work or ideas, but it is also applied to the definition of individual differences in creativity, thus, referring to creativity as the ability to produce ideas that are novel and useful. This conceptualization focuses on creativity as a cognitive ability or potential, but it does not imply other conceptualizations conceiving creativity as personality trait or equating it with actual creative activities or achievements (for a more detailed overview of different conceptualizations of creativity, see Jauk, Benedek, & Neubauer, 2014). The psychometric assessment of this creative ability mainly relies on divergent tasks, which ask for the generation of creative ideas to open problems (e.g., find creative alternative uses for a brick; Kaufman, Plucker, & Baer, 2008). Responses to divergent thinking tasks are then scored for creativity or other characteristics (e.g., fluency, flexibility, and originality). Divergent thinking ability is widely acknowledged as an indicator of the potential for creative thought (Runco & Acar, 2012), and there is evidence for its validity with respect to real-life creativity (e.g., Benedek, Borovnjak, Neubauer, Kruse-Weber, 2014; Plucker, 1999).

The literature has consistently reported a positive relationship between intelligence and creativity (Batey and Furnham, 2006, Kim et al., 2010). According to meta-analytic evidence the average correlation between manifest indicators of these two traits is rather modest (*r* = .17; Kim, 2005). However, much more substantial correlations are usually obtained in studies using latent variables (e.g., Jauk et al., 2014, Silvia, 2008), when creativity is indexed by measures considering the creative quality of generated ideas rather than by ideational fluency (i.e., assessing the number of ideas), and when creativity tasks explicitly require participants to be creative (Nusbaum, Silvia, & Beaty, in press). Moreover, intelligence shows higher correlations with cognitive indicators of creativity (i.e., divergent thinking ability) than with self-report measures of creativity, creative activities, or creative achievements (e.g., Batey et al., 2010, Jauk et al., 2014). Finally, the size of correlation may also depend on the considered facet of intelligence in terms of stratum-II factors of the CHC model of intelligence (Carroll, 1993, McGrew, 2009). High correlations are usually observed with Gf and Gr, but correlations appear to be lower with Gc (Beaty and Silvia, 2013, Cho et al., 2010, Silvia and Beaty, 2012, Silvia et al., 2013).

The average intelligence level of the sample appears to be another important moderator of the intelligence–creativity relationship (Cho et al., 2010, Jauk et al., 2013, Karwowski and Gralewski, 2013). The correlation is often found to be higher in subsamples of lower as compared to higher intelligence, which is referred to as the *threshold-effect*. This threshold effect is thought to imply that intelligence represents a necessary (but not sufficient) precondition of creativity that is relevant up to a certain intelligence level, whereas further increases of intelligence beyond that threshold become less important. A recent study showed that this intelligence-threshold is higher for more demanding indicators of creativity (i.e., ideational creativity vs. ideational fluency; Jauk et al., 2013). For a particularly complex indicator such as creative achievement, however, no threshold was observed anymore, suggesting that intelligence is relevant for creative achievement across the entire IQ range (Jauk et al., 2013, Park et al., 2008; cf. Robertson, Smeets, Lubinski, & Benbow, 2010). It should be noted that the threshold effect is not without controversy with several studies reporting no support for a threshold effect (e.g., Preckel, Holling, & Wiese, 2006; cf. Kim, 2005). In part, failure to observe a threshold effect may be related to the common practice of using fluency-dependent creativity measures and arbitrarily assuming a threshold at IQ = 120. A recent empirical investigation estimated the threshold for ideational fluency to be IQ = 86, whereas the threshold for average originality was estimated at IQ = 119 (Jauk et al., 2013).

But what are the mechanisms that underlie the observed correlation of intelligence and creativity? A number of studies have begun to shed light on the functional role of intelligence in creative thought. Gilhooly, Fioratou, Anthony, and Wynn (2007) performed an analysis of the processes and strategies involved in idea generation based on verbal protocols acquired during task performance. They observed that creative idea generation initially mainly relies on retrieval from memory (cf. Benedek, Jauk, et al., 2014b;), but the generation of novel uses is rather related to more elaborate strategies that occur later in the task. Moreover, the generation of novel (but not of old, known) ideas was associated with letter fluency performance, which is thought to indicate higher involvement of executive processes in the generation of novel ideas. Nusbaum and Silvia (2011b) experimentally tested this notion by instructing half of the participants to use a certain strategy when they get stuck during idea generation (i.e., consider the disassembly of objects during the generation of alternate uses of this object; cf. Gilhooly et al., 2007). They found that Gf predicted the creativity of ideas more strongly in the strategy group than in the control group, suggesting that intelligence facilitates the fruitful implementation of demanding cognitive ideation strategies leading to overall higher creativity. Further evidence comes from a study analyzing the effect of intelligence on creativity over the course of idea generation (Beaty & Silvia, 2012). While the creativity of ideas generally increases (and the fluency of ideas decreases) with time on task, intelligence was associated with higher total creativity and with lower increases over time. More intelligent people thus are more likely to generate creative ideas right from the start, which leaves little room for improvement over time, whereas less intelligent people rather start off with more common, uncreative ideas. Intelligence hence could be related to an effective suppression of interference from dominant, obvious ideas. In a similar vein, high creativity was shown to be related to high dissociation ability and a fast transition from common to uncommon responses in word association tasks, which points to more effective controlled search of memory in creative people (Benedek et al., 2012b, Benedek and Neubauer, 2013). Together, these studies highlight some potential mechanisms of how intelligence and executive processes may facilitate creative thought.

### 1.2 Executive functions

Executive functions (EFs) are basic cognitive processes that control thought and action. EFs are tightly linked to neural substrates in the prefrontal cortex, and they are drawn on for explaining impairments of cognitive control after brain lesions (Miller & Cohen, 2001). Moreover, EFs are thought to be crucially involved in all kinds of higher-order cognition (Miller & Wallis, 2009). Commonly postulated EFs include updating, shifting, and inhibition (Friedman et al., 2006, Miyake et al., 2000). *Updating* is closely associated with the concept of working memory (Jonides & Smith, 1997). It refers to the monitoring of incoming information and the revision of working memory content by replacing obsolete information with information that is new and relevant for the current task. A prototypical updating task is the *n-back* task, which requires a continuous update of working memory in order to maintain the series of the last *n* presented elements of information. *Shifting* refers to the process of switching between different tasks and mental sets (Monsell, 1996). As conditions change, different rules and responses may become appropriate. Shifting involves the disengagement of a mental set that has become irrelevant set in favor of the engagement of a new and relevant mental set or task. A common task to assess shifting is the number–letter task (Rogers & Monsell, 1995) that requires either making odd–even decisions or consonant–vowel decisions depending on the position of the stimulus. The EF *inhibition* can be defined as the suppression of dominant but irrelevant response tendencies. A well-known inhibition task is the Stroop task which requires inhibiting the tendency of producing an automatic response such as naming the stimulus word (Stroop, 1935). It should be noted that the concept of inhibition is particularly diverse, and may, in different contexts, also denote other conceptualizations such as the control of distractor interference (Friedman & Miyake, 2004).

In an influential work by Miyake et al. (2000), the authors examined the unity and diversity of the EFs updating, shifting, and inhibition. The three EFs were found to be substantially correlated, but there was also factor-analytic evidence for their independence (cf. Friedman et al., 2008, Miyake and Friedman, 2012). The authors further examined the role of EFs in the performance of complex mental tasks employing an individual differences approach. They showed that updating, shifting and inhibition contribute differentially to more complex (executive) tasks such as the Tower of Hanoi or the Random Number Generation Task. The findings suggest that individual differences in higher-level cognition may be traced back to individual differences in executive abilities, and that the relevance of different EFs may vary depending on the considered task or construct.

### 1.3 Intelligence and executive functions

Research on the relationship of intelligence and executive functions has clearly focused on updating or working memory (cf. Conway, Getz, Macnamara, & Engel de Abreu, 2010). Numerous studies consistently reported substantial positive correlations between measures of intelligence and working memory capacity (Ackerman et al., 2005, Colom et al., 2008, Conway et al., 2003, Oberauer et al., 2008, Shelton et al., 2009). Using latent variable analysis, these relationships sometimes approached a perfect correlation suggesting that g and working memory might even be the same constructs (Ackerman et al., 2005, Bühner et al., 2005, Colom et al., 2004). A meta-analysis revealed an average correlation of .48 between intelligence and working memory capacity, indicating that intelligence and working memory are fairly correlated but still distinguishable at the manifest level (Ackerman et al., 2005). Re-analyses using a latent variable approach showed that the average latent correlation between working memory and fluid intelligence is markedly higher but still not perfect (Kane et al., 2005, Oberauer et al., 2005, Shelton et al., 2009). Working memory and intelligence thus can be seen as highly correlated constructs that, however, are not isomorphic.

There exists considerably less research and evidence on the relationship of intelligence with other EFs besides working memory. One study by Friedman et al. (2006) examined how intelligence relates to updating, shifting, and inhibition using a similar latent variable design as in Miyake et al. (2000). They found that only updating significantly predicted both fluid and crystallized intelligence, but inhibition and shifting did not. The authors concluded that current intelligence tests assess only a part of the executive abilities that are involved in cognitive control. Taken together, research on intelligence and EFs suggest that intelligence is highly correlated with updating but probably not with other EFs.

### 1.4 Creativity and executive functions

Theories of creativity stress the importance of avoiding common paths, being able to consider and recombine multiple unrelated concepts, and showing flexibility of perspective (Koestler, 1964, Mednick, 1962, Nijstad et al., 2010). This suggests that EFs like inhibition, updating, and shifting may be relevant for creative thought. Further support of this notion has been inferred from the relationship of creativity with intelligence. But how do different EFs relate to creativity? Early conceptions of creativity stated that “creative people are characterized by a lack of both cognitive and behavioral inhibition” (Martindale, 1999, p. 143; see also, Eysenck, 1995). This notion may be related to the observation that creative people fluently generate ideas and associations (Benedek et al., 2012b, Mednick et al., 1964), and seem to show overinclusive thinking and decreased filtering of task-irrelevant information (i.e., latent inhibition; Carson, Peterson, & Higgins, 2003). Available evidence from studies employing actual measures of inhibition ability, however, rather points in the opposite direction. Studies assessing inhibition by performance in the Stroop task generally reported positive correlations of inhibition with divergent thinking performance and teacher ratings of high school students (Edl, Benedek, Papousek, Weiss, & Fink, in press; Golden, 1975; Groborz & Necka, 2003). Other studies measuring inhibition by the ability to avoid repetitive responses in the random motor generation task also found a positive correlation of inhibition with ideational fluency and self-report indicators of creative behavior and creative achievement (Benedek et al., 2012a, Zabelina et al., 2012). It should be noted that some studies obtained negative correlations of creativity measures with tasks requiring inhibition of interference but positive correlations in tasks without interference (Dorfman et al., 2008, Kwiatkowski et al., 1999, Vartanian et al., 2007), which was interpreted in terms of an adaptive or flexible engagement of inhibition (Vartanian, 2009, Zabelina and Robinson, 2010).

Interestingly, there is not as much empirical evidence on the relationship between creativity and working memory. Two studies reported positive correlations of working memory with creativity assessed by divergent thinking tasks that were scored for fluency or originality (de Dreu et al., 2012; Oberauer et al., 2008), whereas one study reported no significant association (Lee & Therriault, 2013). Another study showed that verbal and visuo-spatial working memory predicts insight and non-insight problem solving ability (Gilhooly & Fioratou, 2009); however, it has recently been questioned whether insight tasks actually tap creativity (Beaty, Nusbaum, & Silvia, in press).

The relationship between creativity and executive shifting seems to be supported by the general consensus that creativity requires flexibility of thought (Chi, 1997). This notion is even built-in in some common divergent thinking tests that score for flexibility of ideation by counting the number of different semantic categories tapped during idea generation (e.g., Torrance, 1974). However, higher ideational flexibility cannot serve as independent evidence for creativity and flexibility at the same time, and independent studies relating creativity with shifting appear to be missing. Support for a relationship of creativity and flexibility of thought mainly comes from intervention studies suggesting that e.g. the induction of positive mood increases both cognitive flexibility and creative problem solving (Ashby et al., 1999, Rowe et al., 2007). Summarizing the available evidence on the relationship of creativity and executive abilities, the findings tend to support a positive association of creativity with inhibition and updating, but evidence is unclear for shifting ability.

### 1.5 Aims of this study

The positive correlation between intelligence and creativity is well documented. However, while intelligence is known to be substantially related to working memory, the executive functions involved in creativity are less clear. The main aim of this study hence is to examine the relative contributions of different EFs to individual differences in (fluid) intelligence and creativity. In addition, we wanted to test the hypothesis that the structural relation between intelligence and creativity is at least partly attributable to individual differences in executive abilities. To this end, we measured intelligence and creativity together with the well-established EF-facets updating, shifting, and inhibition and analyzed their mutual relationships within a latent variable framework.

## 2 Methods

### 2.1 Participants

A total of 243 people participated in this study. Because of the study's substantial language component, we excluded participants who did not speak German as native language (*n* = 5); seven additional participants were excluded due to extensive missing data. This left a final sample of 230 participants (70% females) with an average age of 23 years (*SD* = 3.5; range from 18 to 45). All participants gave written informed consent. The procedure was approved by the Ethics Committee of the University of Graz.

### 2.2 Material and methods

#### 2.2.1 Assessment of executive functions

We assessed three different types of executive functions – updating, shifting, and inhibition – that were previously considered as particularly relevant for complex mental tasks (Friedman et al., 2006, Friedman et al., 2008, Miyake et al., 2000). Moreover, tasks and scoring methods were kept similar as in Miyake et al. (2000).

##### 2.2.1.1 Updating

Updating was assessed by means of a nonverbal 2-back task (Schellig, Schuri, & Arendasy, 2011). The computer-based task presented a total of 100 abstract black figures on white background at a regular pace of 1.5 s per figure. Participants had to decide whether the current figure is identical with the one presented two stimuli ago by pressing a button for each target. The abstract figurative material intends to avoid the use of common verbal rehearsal strategies. The test was found to conform to a 1PL Rasch model (Schellig et al., 2011). The test was split into three blocks of 33 items (excluding the first item), and for each block the performance was scored as the number of correct responses (hits and correct rejections). Internal consistency of scores across the three task blocks was high (Cronbach's α = .89).

##### 2.2.1.2 Shifting

Shifting was assessed by means of the number–letter task (Rogers & Monsell, 1995). In this task, number–letter pairs (e.g., “8G”) were presented in one of the four quadrants of the computer screen in a clockwise order. Participants were required to switch between two subtasks: They were asked to indicate whether the number was odd or even when the stimulus was presented in one of the upper two quadrants, and to indicate whether the letter was a consonant or vowel when it was in one of the bottom two quadrants. They thus had to switch tasks in half of the trials (i.e., trials from the upper left and lower right quadrants). Participants first completed two no-shifting blocks (24 trials each) with instructions focusing exclusively on either number or letter judgments, followed by three shifting blocks (24 trials each). The shift cost for each shift block was defined as the difference between the average reaction time in shift trials and the average reaction time in the no-shift blocks (cf., Miyake et al., 2000). The shifting cost in this task is considered a reverse indicator of shifting ability. Internal consistency of scores across the three task blocks was good (Cronbach's α = .79).

##### 2.2.1.3 Inhibition

Inhibition of prepotent responses was measured with the Stroop color–word-interference task (Stroop, 1935). The Stroop task is often thought to be a prototypical inhibition task (Miyake et al., 2000). The task presented single words denoting either a color name (“red”, “green”, “blue”, or “yellow”), or “XXXX” on a black computer screen. The color of the stimuli was either *congruent* or *incongruent* to its meaning; in the case of “XXXX” the coloring was considered neutral with respect to its meaning. Participants were asked to name the color of the stimuli as fast as possible (time-out = 4 s) by entering one out of four keys associated with the color. For incongruent trials, this required inhibiting the dominant process of naming the word. Participants received a feedback for incorrect responses to ensure high accuracy. The task included one practice block, and three further task blocks. Each block consisted of 32 trials: 16 neutral, 12 incongruent, and 4 congruent trials. The Stroop effect was scored for each block as the difference of the mean reaction time in incongruent and neutral trials. The Stroop effect is considered a reverse indicator of inhibition. Internal consistency of scores across the three task blocks was satisfactory (Cronbach's α = .61).

#### 2.2.2 Assessment of intelligence

Intelligence was assessed by means of two subtests of the intelligence structure battery (INSBAT; Arendasy et al., 2008), which were constructed to measure fluid intelligence (Gf). We selected the subtests numerical-inductive reasoning (NID; Arendasy & Sommer, 2012) and verbal-deductive reasoning (VDD; Arendasy et al., 2008). The two subtests were constructed by means of automatic item generation (AIG: Arendasy and Sommer, 2011, Arendasy and Sommer, 2012, Irvine and Kyllonen, 2002) on the basis of theoretical models on inductive and deductive reasoning. The numerical-inductive reasoning subtest requires participants to discover the rules which govern number series while the verbal-deductive reasoning test consists of syllogism tasks. Both subtests were calibrated by means of the 1PL Rasch model (Rasch, 1980). Previous studies indicated that both subtests exhibit a high g-factor saturation (e.g., Arendasy et al., 2008a, Arendasy and Sommer, 2012, Arendasy et al., 2008b) and item design features linked to cognitive processes involved in inductive and deductive reasoning account for up to 88% of the variance in the 1PL item and person parameters. Both subtests were presented as computerized adaptive tests (CATs) with a target reliability corresponding to α = .80. Test administration took on average 15 min for NID and 10 min for VDD. All participants in the present study reached the predefined level of measurement precision.

#### 2.2.3 Assessment of creativity

Creativity can be conceived to involve different components such as creative potential, expertise, relevant personality traits, and actual creative behavior (e.g., Amabile, 1983). In this study, we focused on creativity limited to creative potential (or creative ability) which is expected to show the closest association with executive functions. For reasons of simplicity and consistency with other similar research (Nusbaum and Silvia, 2011a, Nusbaum and Silvia, 2011b, Silvia et al., 2013; cf. Silvia, Winterstein, & Willse, 2008), we will use the term *creativity* in this manuscript when referring to the ability component of creativity (i.e., creative potential, or divergent thinking ability), while still acknowledging the multi-facetted nature of the creativity construct.

The predominant approach to the psychometric assessment of creative potential is by means of divergent thinking (DT) tasks (Kaufman et al., 2008, Runco and Acar, 2012). We used four DT tasks including two alternate uses tasks (“What can a tin can be used for?”, “What can a car tire be used for?”) and two instances tasks (“What can be round?”, “What can be used for speedy travel?”). Tasks were administered on a PC, and participants had 2 min per task to name all the creative responses that they could think of. All responses were rated for creativity by four experienced raters on a four-point scale ranging from ranging from 1 (not creative) to 4 (very creative). Raters were told that creativity evaluations should reflect both originality/unusualness and appropriateness of the idea in a single holistic judgment (e.g., Silvia et al., 2008), and that high creativity ratings should only be assigned to ideas that only few people could presumably come up with. Interrater-reliability in the four DT tasks was good (ICC = .74, .79, .57, and .72). The tasks were finally scored for creativity by means of the top-scoring method (Benedek et al., 2013, Silvia et al., 2008b), which was shown to avoid a confounding with the number of responses (Benedek et al., 2013, Silvia et al., 2008b). For each task, creativity scores reflected the average creativity rating of those three ideas that had received the highest ratings from the raters.

#### 2.2.4 Personality

We assessed the individual personality structure by means of the Big-Five personality test NEO-FFI (Borkenau & Ostendorf, 1993). The NEO-FFI contains a total of 60 items and was administered on PC.

### 2.3 Procedure

Participants were tested in groups of up to 6 people. After completing an informed consent form, participants completed the divergent thinking tasks (alternate uses and instances), the intelligence tasks (NID and VDD), and the updating task. After a short break, they completed the inhibition and shifting tasks, and self-report questionnaires of creativity. All psychometric tests were administered on PC either using the Vienna Test System (VTS; Schuhfried, 2013) or Matlab software (The MathWorks; Natick, MA). The total session took about 100 min.

## 3 Results

### 3.1 Descriptive statistics and model specification

Latent variable models were used to estimate latent relationships between executive functions, fluid intelligence, and creativity. There was little missing data: covariance coverage was at least 94% and was typically 98% to 100%. All models were estimated with MPlus 7 (Muthén & Muthén, 1998–2012). For some measures there was evidence for moderate non-normality (skew < 2, kurtosis < 7); although ML estimators are fairly robust to these conditions, models were estimated using the maximum likelihood procedure with robust standard errors (MLR) which is specifically robust in face of non-normality of the data (Finney and DiStefano, 2006, Muthén and Muthén, 1998–2012). Table 1 presents descriptive statistics and the inter-correlations of all measures. The total scores of all factors (fluid intelligence, creativity, and the three executive functions) were tested for sex differences. We did not observe sex differences in fluid intelligence (*t*[228] = − 1.42, *p* = .16), updating (*t*[228] = − 1.64, *p* = .10), shifting (*t*[223] = − 1.38, *p* = .17), or inhibition (*t*[223] = 0.06, *p* = .95); however, men showed higher creative potential than females (*t*[224] = − 3.60, *p* < .001, *d* = 0.52).

**Table 1:** Descriptive statistics and correlations.

	*M*	*SD*	1	2	3	4	5	6	7	8	9	10	11	12	13	14	15	16	17
1 N-back B1	28.20	3.84	–																
2 N-back B2	27.75	4.49	.72	–															
3 *N*-back B3	27.84	4.77	.66	.83	–														
4 Number–letter B1	1.50	0.77	− .05	− .09	− .05	–													
5 Number–letter B2	1.07	0.70	.03	.00	− .02	.61	–												
6 Number–letter B3	0.92	0.66	.11	.06	.07	.50	.70	–											
7 Stroop B1	0.24	0.21	.01	.04	.00	− .07	− .01	− .08	–										
8 Stroop B2	0.19	0.18	.10	.08	.10	− .11	.01	− .08	.34	–									
9 Stroop B3	0.20	0.20	.04	.06	.11	− .04	.00	− .04	.38	.24	–								
10 NI reasoning	1.04	1.66	.28	.32	.33	− .15	− .09	− .02	.07	.00	.04	–							
11 VD reasoning	0.97	1.10	.22	.27	.24	.04	.00	.02	.11	.04	.07	.34	–						
12 Uses: tin can	1.33	0.30	.13	.16	.16	− .03	− .05	− .12	.09	.07	.02	.15	.04	–					
13 Uses: car tire	1.20	0.27	.14	.16	.18	.10	.04	− .06	.08	.03	.06	.12	.17	.36	–				
14 Instances: round	1.28	0.23	.12	.11	.10	.15	.04	− .02	.15	.02	.09	.23	.21	.37	.31	–			
15 Instances: travel	1.17	0.25	.23	.22	.22	.08	.04	− .01	.09	− .02	.08	.17	.11	.31	.32	.39	–		
16 Openness P1	10.10	2.66	.06	.08	.02	− .07	− .04	− .11	.05	.02	− .04	.12	.19	.23	.26	.15	.13	–	
17 Openness P2	11.19	2.25	.05	.10	.03	− .08	− .01	− .07	.10	− .09	.02	.03	.02	.19	.25	.11	.17	.46	–
18 Openness P3	11.07	2.84	.05	.11	.06	− .11	− .10	− .15	.07	.04	.07	.08	.11	.22	.21	.13	.09	.66	.47

Notes. The correlation matrix was adjusted for missing data by MPlus. For reaction time measures (i.e., number–letter and Stroop), scores were reversed so that higher values indicate better (faster) performance. NI = numerical-inductive, VD = verbal deductive, B = block, P = parcel; *p* < .05 for *r* ≥ .13, and *p* < .01 for *r* ≥ .17 given that *n* = 230.

The latent variable *updating* was defined by three task blocks of the 2-back task, the latent variable *shifting* was defined by three task blocks of the number–letter task, and the latent variable *inhibition* was defined by three task blocks of the Stroop task. Note that indicators of the number–letter task and the Stroop task were reversed so that better task performance indicates higher shifting or inhibition ability, respectively. *Fluid intelligence* (Gf) was defined by scores in the numerical-inductive reasoning task (NID) and in the verbal-deductive reasoning task (VDD), and *creativity* was defined by rated creativity scores in the four divergent thinking tasks. Finally, for separate analyses including the latent variable openness, *openness* was defined by three item parcels each aggregating four different items. In each measurement model the first indicator was fixed to 1. All models were also run separately with sex as manifest exogenous variable relating to all latent variables. Including sex, however, had no visible effect on the structural relations in these models and the variable sex was not included in the reported models. All estimates were standardized.

### 3.2 Executive abilities, intelligence and creativity

In order to ensure adequate measurement of the latent constructs before testing the structural relationships among them, we followed a two-step modeling approach (Anderson & Gerbing, 1988). In a first step, we specified two measurement models, one for executive abilities (i.e., predictor part of the final model) and one for the cognitive abilities fluid intelligence and creativity (i.e., the criterion part of the final model). The fit of the measurement model of executive abilities was very good: χ^2^(30) = 22.54, *p* = .57; CFI = 1.000, RMSEA = .00 (90% CI: .00, .05); SRMR = .034. No significant correlations were observed between latent factors of updating, shifting and inhibition. The fit of the measurement model for fluid intelligence and creativity was also very good: χ^2^(12) = 13.74, *p* = .32; CFI = .989, RMSEA = .025 (90% CI: .00, .07); SRMR = .031. The general latent correlation between Gf and creativity was .45 (*p* < .001).

**Fig. 1:**
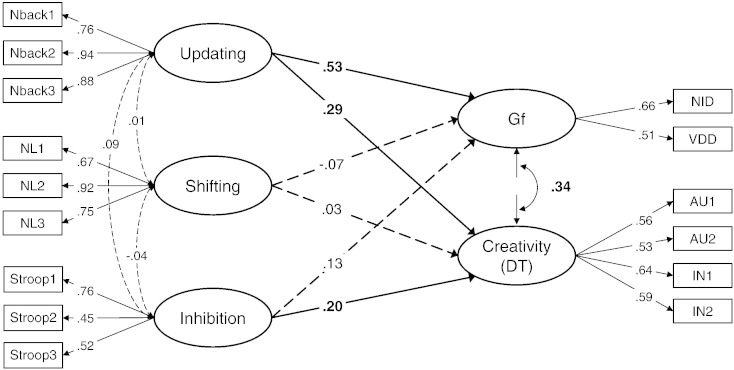
Latent variable model with executive abilities (updating, shifting and inhibition) predicting fluid intelligence and creativity (i.e., divergent thinking ability). Scores are reversed for reaction time measures, i.e., number–letter and Stroop task, so that higher values indicate better performance. Dotted lines indicate non-significant paths. NL = number–letter task, NID = numerical-inductive reasoning task, VDD = verbal deductive reasoning task, Gf = fluid intelligence, DT = divergent thinking, AU = alternate uses task, IN = instances task.

Next, we examined how different executive functions predict Gf and creativity by regressing them on latent variables of updating, shifting, and inhibition with all paths freely estimated. Furthermore, we allowed the latent residuals of Gf and creativity to be correlated to test whether Gf and creativity are correlated even after accounting for variance in both latent traits that is attributable to EFs. This model fitted the data well: χ^2^(80) = 81.51, *p* = .43; CFI = .998, RMSEA = .009 (90% CI: .00, .04); SRMR = .041. Fig. 1 displays the standardized factor loadings and path coefficients of this model. The inter-correlations between the latent executive functions were all non-significant. The model further revealed that updating strongly predicts Gf (β = .53, *p* < .001) and, to a lesser extent, also creativity (β = .29, *p* < .001). By contrast, inhibition predicted creativity (β = .20, *p* = .04) but not Gf (β = .12, *p* = .30). Shifting did not show any significant relations with Gf or creativity. The residual correlation of Gf and creativity in this model was .34 (*p* = .01). Dropping the non-significant paths from shifting to Gf and creativity and from inhibition to Gf did not reduce the model fit (Santorra–Bentler scaled Δχ^2^ = 2.14, *p* = .54) and the standardized estimates of all path coefficients remained essentially unchanged.

### 3.3 Explaining the relationship of intelligence and creativity

The previous model already indicated that executive functions can explain a part of the common variance of fluid intelligence and creativity, but a significant residual correlation was still observed. To further explore the remaining correlation of Gf and creativity, we examined the role of personality. Only openness to experience showed significant zero-order correlations to both Gf and creativity. Openness was thus included as another predictor to the model. This extended model again showed a very good fit: χ^2^(120) = 124.06, *p* = .38; CFI = .996, RMSEA = .012 (90% CI: .00, .04); SRMR = .042. In this model, openness predicts creativity (β = .36, *p* < .001) but not Gf (β = .16, *p* = .15). By adding openness as a predictor the residual correlation of Gf and creativity further was reduced and now showed only a tendency toward a positive correlation (*r* = .29, *p* = .06).

## 4 Discussion

### 4.1 What executive functions are related to intelligence and creativity?

The main aim of this study was to examine the role of the executive functions (EFs) updating shifting and inhibition for intelligence and creativity. Fluid intelligence was significantly predicted by updating but neither shifting nor inhibition showed significant associations. This finding is in line with the literature which consistently reports a substantial positive relationship of updating ability or working memory with intelligence (Ackerman et al., 2005, Bühner et al., 2005, Colom et al., 2004, Colom et al., 2008, Conway et al., 2003, Kane et al., 2005, Oberauer et al., 2008). Furthermore, the results also replicate previous findings by Friedman et al. (2006) showing that inhibition and shifting do not explain significant unique variance of intelligence beyond updating.

Turning to creativity, both updating and inhibition significantly predicted creativity (i.e., divergent thinking ability) but shifting did not. Creativity was previously found to be correlated with inhibition defined either by performance in the Stroop task (Golden, 1975, Groborz and Necka, 2003) or the random motor generation task (Benedek et al., 2012a, Benedek et al., 2012b, Zabelina et al., 2012). Prepotent response inhibition is thought to facilitate creative thought by suppressing interference caused by dominant response tendencies (Benedek et al., 2012a, Benedek et al., 2012b, Gilhooly et al., 2007). In the context of creative idea generation, dominant responses reflect obvious, common ideas that are highly salient but not creative. The generation of creative ideas, however, likely requires the activation and retrieval of concepts that are only remotely associated with the problem or stimulus at hand. The selective retrieval of relevant but weakly related concepts hence is supported by the effective inhibition of salient, strongly related concepts (Friedman and Miyake, 2004, Gupta et al., 2012).

Interestingly, updating showed an even slightly higher association with creativity than inhibition. Working memory is a primary resource for the control of attention (e.g., Engle, 2002; Kane, Bleckley, Conway & Engle, 2001). It supports the active maintenance of task-relevant information and the controlled search from memory (Unsworth & Engle, 2007). As mentioned before, creative ideas originate from the successful association of previously unrelated concepts taken from memory (Koestler, 1964, Mednick, 1962). Considering the alternate uses task, a common divergent thinking task, this task requires the generation of creative novel uses for common objects (e.g., a car tire). Fertile strategies for idea generation involve the identification of relevant parts or properties of the object (e.g., size and shape), and the search of possible applications related to those characteristics but not directly related to the stimulus object (e.g., “use it as picture frame”). The generation of responses that fulfill those criteria hence requires controlled search and selective retrieval from memory. In this context, working memory is involved in the identification and maintenance of relevant cues that help delimiting the actual search set (Unsworth & Engle, 2007). Higher working memory capacity hence facilitates a more effective search of memory, leading to a higher likelihood of retrieving relevant semantic concepts that qualify for creative ideas (cf. Wiley & Jarosz, 2012).

Working memory is also responsible for the active maintenance of task goals. Again considering the alternate uses task, this task implies the goal to generate uses for objects, requires that responses are creative, and it may involve additional goals derived from specific idea generation strategies (cf. Gilhooly et al., 2007). People with higher working memory capacity may more easily keep all goals active throughout the task, whereas people with lower working memory capacity may fall back on less specific goals (e.g., generating uses that are retrieved from memory and thus are likely uncreative). This interpretation is supported by recent findings. In a study using a long brainstorming task, working memory capacity was shown to be positively associated with ideational fluency (within semantic categories and in total) and average originality during a task which was interpreted in terms of higher persistence in task performance (de Dreu et al., 2012). Another study showed that the explicit instruction to focus on the creativity of ideas increased the influence of intelligence on creative performance as compared to the less demanding instruction to focus on generating a high number of ideas (Nusbaum et al., in press). Similarly, intelligence was shown to moderate the effectiveness of using a specific idea generation strategy (i.e., focus on object properties), consistent with the notion that active maintenance of such a strategy depends on working memory capacity (cf. Nusbaum & Silvia, 2011b).

Cognitive neuroscience provides an additional perspective on the role of attention in divergent thinking (i.e., creative idea generation). Divergent thinking tasks such as the alternate uses task were shown to represent top-down activity characterized by focused internal attention (Benedek et al., 2011, Benedek et al., 2014). In DT tasks, masking of the stimulus neither affects task performance nor brain activation. In contrast, for tasks which are dependent on sensory information, stimulus masking leads to lower task performance and a similar brain activation pattern as during divergent thinking, which is typically characterized by increased right-parietal alpha synchronization in the EEG (Fink and Benedek, 2013, Fink and Benedek, in press) and reduced activation of the ventral salience network in fMRI (Benedek et al., 2014a, Benedek et al., 2014b, Fink et al., 2009). It was proposed that focused internal attention plays an important role of task-shielding during creative idea generation which may be particularly relevant during processes of imagination and mental simulation (Benedek et al., 2014b, Benedek et al., 2014c). These processes may involve the generation of mental images and thus be especially sensitive to distracting external stimulation.

Common theories of creativity assume that the generation of creative ideas requires the adequate recombination of unrelated semantic concepts (Koestler, 1964, Mednick, 1962). The finding that inhibition and updating both predict creativity appears to be well in line with those theories. Inhibition reflects the ability to suppress interference by semantically close concepts and thus facilitates the activation of semantically remote concepts. High updating ability or working memory capacity facilitates the controlled search and manipulation of a larger number of concepts. These two executive abilities hence may fruitfully act together in the generation for creative thought. Previous empirical support for those theories came see changes from a word association study showing that creativity was related to *dissociative ability* and the ability of *associative combination* (Benedek et al., 2012a, Benedek et al., 2012b), which seem to tap similar cognitive mechanisms. Interestingly, *associative flexibility* did not explain further variance of creativity in that study, just as shifting did not in the present study.

These considerations are thought to outline some of the cognitive mechanisms of how executive abilities may be involved in creative thought. Taken together, this study provides further support for the executive nature of creativity (Beaty and Silvia, 2012, Benedek and Neubauer, 2013; Nusbaum et al., in press). According to this view, creative thought does not solely depend on spontaneous thought processes, but strongly relies on controlled top-down activity (cf. Abraham, 2014, Beaty et al., in press).

### 4.2 Do executive abilities explain the correlation of intelligence and creativity?

Intelligence and creativity as measured by divergent thinking tasks showed a latent correlation of *r* = .45 in this study, which is nearly identical to the correlations typically observed in latent models of intelligence and creativity (cf. Beaty and Silvia, 2013, Benedek et al., 2012a, Benedek et al., 2012b, Nusbaum and Silvia, 2011b, Silvia et al., 2013). Including the EFs updating, shifting, and inhibition as predictors to the model resulted in a reduced but still significant residual correlation between intelligence and creativity (*r* = .34), corresponding to reduction of shared variance by 43%. This reduction can be essentially attributed to updating, which significantly predicted both intelligence and creativity. Adding the personality factor openness to the model further slightly reduced the residual correlation of intelligence and creativity, now leaving only a correlation by trend (*r* = .29). These findings indicate that updating ability represents the central executive mechanism underlying the correlation of intelligence and creativity. Moreover, individual differences in updating and openness can together explain a relevant part but probably not the entire correlation between intelligence and creativity.

In the previous sections we have already outlined some possible cognitive mechanisms of how updating is involved in intelligence and creativity. Considering openness, this personality factor is consistently related to intelligence (Ackerman and Heggestad, 1997, DeYoung, 2011) as well as to creativity (Feist, 1998; McCrae, 1987). It should be noted that recent studies suggest a more differentiated view on the relationships between openness, intelligence, and creativity. First of all, openness typically shows stronger correlations with crystallized rather than fluid intelligence (Ashton et al., 2000). Moreover, within the conceptual framework dividing the openness into two aspects of openness and intellect (DeYoung, Quilty, & Peterson, 2007), intelligence appears to be particularly related to the intellect aspect (DeYoung, Quilty, Peterson, & Gray, 2014), whereas creativity shows higher correlations with the openness aspect (Kaufman, 2013, Nusbaum and Silvia, 2011a). However, different aspects of the openness construct also share some common mechanisms, which likely include the drive to seek and explore new information. Moreover, openness is conceived as an investment trait that fosters cognitive abilities including creative potential via curiosity and increased engagement in various intellectual activities (Ackerman, 1996, Chamorro-Premuzic and Furnham, 2005, von Stumm et al., 2011, Ziegler et al., 2012). Such general personality–ability mechanisms thus might contribute to the shared variance between intelligence and creativity.

### 4.3 Limitations and future directions

A few limitations of the present study should be acknowledged. First of all, this study used latent variables of executive functions, which were each defined by three task blocks of a relevant executive task, but not by different tasks of the same construct. This approach allows the definition of reliable and homogeneous latent executive factors without running the risk of including tasks of unclear validity (cf. Salthouse, Atkinson, & Berish, 2003). Evidence for the validity of this approach was obtained from the observation that the relationships of executive functions and intelligence replicated the results of previous studies using more broadly defined factors (e.g., Ackerman et al., 2005, Friedman et al., 2006). However, although the selected tasks are often regarded as gold standards for the assessment of updating, inhibition and switching, the most adequate assessment of those constructs as well as their convergent and discriminant validity is still up for debate (cf. Salthouse et al., 2003). Findings thus should be interpreted with caution. Moreover, the employed procedure does not account for method variance specific to the task. Therefore, latent correlations may have been underestimated in this study, which might explain why the three EFs updating, shifting, and inhibition were essentially uncorrelated in this study, although they showed substantial inter-correlations in other studies (Friedman et al., 2006, Miyake et al., 2000). It should be noted, however, that in those studies, substantial inter-correlations between EFs were only observed at latent level, whereas the zero-order correlations of tasks tapping different EFs were generally close to zero. In any case, future research may aim at replicating findings with more broadly defined executive factors in order to increase the generalizability of interpretations.

Following previous similar studies, inhibition was conceptualized as prepotent response inhibition in this study (cf. Friedman et al., 2006, Miyake et al., 2000). However, the construct of inhibition is quite diverse and sometimes also refers to different conceptualizations in the literature. For instance, inhibition may also refer to concepts such as the *resistance to distractor interference* or to *resistance of proactive interference* (Friedman & Miyake, 2004). While the functional relationship between prepotent response inhibition and creativity appears quite straightforward, it would also be interesting to consider the role of other types of inhibition in future research. Effective suppression of proactive interference, for example, could play a role in creative idea generation by reducing interference of initial ideas and thus avoiding tendencies to get stuck or perseverate. Moreover, reduced latent inhibition paired with high intelligence could enable the perception and integration of a larger amount of potentially relevant stimuli during creative thought (Carson et al., 2003). A final limitation can be seen in the lack of sex balance in our sample. Although a higher representation of females is quite common in psychological studies, it may still limit generalizability.

The consistent relationship between intelligence and creativity suggests an executive nature of creativity. The present findings demonstrate that this relationship can be mainly attributed to individual differences in updating ability. We have outlined some possible mechanisms of how updating and inhibition may facilitate creative thought. Future research may continue to explore these potential mechanisms more directly. This could be done, for example, by testing how individual differences in specific executive abilities are related to the recruitment and successful implementation of cognitive strategies and memory search processes during creative thought.
